# Prevalence of overweight and obesity among preschool children in Hainan: a cross-sectional study in China's largest free-trade zone

**DOI:** 10.3389/fped.2024.1476231

**Published:** 2025-01-07

**Authors:** Wei-Jia Wu, Ping-Hao Chen, Zhen-Ning Huang, Xue-Lu Lei, Chen Wang, Chun-hui Zhang, Ping Wang, Chui-can Huang, Qing Luo, Li-Chun Fan

**Affiliations:** ^1^ Hainan Women and Children’s Medical Center, Hainan Medical University, Hainan Academy of Medical Sciences, Haikou, China; ^2^ School of Pediatrics, Hainan Medical University, Haikou, China; ^3^ School of Basic Medicine and Life Science, Hainan Medical University, Haikou, China; ^4^ School of Public Health, Hainan Medical University, Haikou, China

**Keywords:** overweight, obesity, preschool children, WHO criteria, IOTF criteria, risk factors

## Abstract

**Background and aims:**

Childhood obesity leads to significant health risks, emphasizing the critical need for effective preventive measures during the preschool years. However, there is a lack of comprehensive studies on overweight and obesity among preschool children in Hainan Island, China's largest free-trade zone. Our study assessed the prevalence of overweight and obesity among preschool children across Hainan Island using Chinese, World Health Organization (WHO), and International Obesity Task Force (IOTF) criteria. Additionally, the potential factors influencing overweight and obesity among preschool children in Hainan Island were further explored.

**Methods:**

We conducted a cross-sectional survey of children aged 3–6 years covering 18 cities and counties in China's largest free trade zone. The survey primarily involved anthropometric data collection and questionnaires on children's information and the sociodemographic characteristics of their parents or guardians. We recorded the height and weight of each child and calculated their body mass index (BMI). The z-score for BMI-for-age was calculated using the WHO-recommended Anthro and AnthroPlus software, and diagnoses of overweight and obesity were determined separately according to the Chinese, WHO, and IOTF criteria. The chi-squared test, *t*-test, or rank-sum test was applied to describe and statistically analyze the baseline characteristics of the participating children. Additionally, potential factors affecting overweight and obesity were analyzed using a multifactor binary logistic regression model.

**Results:**

The prevalence of overweight and obesity among preschool children in Hainan Island was 11.30% based on the Chinese criteria, significantly higher than the rates of 5.80% and 5.40% observed according to the WHO and IOTF criteria, respectively (*χ*^2^ = 12,870.368, *P* *<* 0.001). After multifactorial adjusted analyses, we found that according to the three growth criteria, having overweight fathers or mothers, family incomes of more than 100,000 Yuan, fully active eating, and higher birth weights increased the risk of overweight and obesity among preschool children in Hainan Island.

**Conclusion:**

The prevalence of overweight and obesity among preschool children in Hainan Island was lower compared to rates reported in other regions. Considering that the growth and development indicators for children under 7 years old in Hainan Island are generally lower than international standards, the use of Chinese standards may be more suitable for detecting overweight and obesity among preschool children in this area. Based on the risk factors identified in this study, preventive measures can be targeted in the future to reduce the risk of overweight and obesity in preschool children in Hainan Island.

## 1 Introduction

The World Health Organization (WHO) defines overweight and obesity as an abnormal or excessive accumulation of fat, which constitutes a health risk (1). The prevalence of childhood obesity has reached pandemic proportions, placing a heavy burden on the global public health system (2). According to reliable data, the age-standardized prevalence of obesity among children and adolescents aged 5–19 years remarkably increased globally from 1975 to 2016, ranging from 0.7% to 5.6% for girls and from 0.9% to 7.8% for boys (1). This trend is also evident in China, where the prevalence of childhood overweight increased from 17.1% to 19.1% between 2009 and 2019, and the prevalence of childhood obesity increased from 11.9% to 12.4% (3).

Childhood obesity may lead not only to stigmatization in society and bullying at school but also to a decline in academic performance and mental health problems. Moreover, it considerably increases the risk of certain types of cancer, metabolic syndrome, type 2 diabetes, bone and joint diseases, and many cardiovascular diseases (1). In addition, childhood obesity is an independent predictor of overall mortality and cannot be ignored (4). Adiposity rebound in children occurs between the ages of 3 and 6 years, and an earlier age of adiposity rebound may notably increase the risk of obesity in adulthood. Therefore, active and effective preventive measures to control weight before the period of adiposity rebound are critical for preventing childhood obesity (5). Preschool years are a critical stage for the development of obesity-related eating and physical activity habits, and many unhealthy habits tend to develop during this period (6). The development and implementation of targeted prevention and intervention programs for preschool children (aged 3–6 years) will be crucial in curbing the spread of childhood obesity (7, 8).

The complexity of the growing problem of overweight and obesity in children stems from the intertwined influences of biological, socioeconomic, and environmental factors (9). These multiple factors interact to promote the excessive deposition and proliferation of adipose tissue (1). Notably, the prevalence of obesity varies by sex, age, and geographic location (10). In China, rapid economic growth, profound sociocultural changes, and policy orientation have led to rapid urbanization. In this process, people's dietary habits have changed remarkably, with a substantial increase in the consumption of animal foods, refined grains, and processed foods that are high in sugar and fat content. Simultaneously, sedentary lifestyles have become prevalent, while the amount of time spent on physical activity has rapidly declined. These shifts in eating habits and lifestyles are intertwined with individual risk factors, such as genetic predispositions, psychosocial stresses, and environmental exposures during the fetal period and early life, which exacerbate the magnitude of the problem of overweight and obesity in children (4).

The free-trade port policy has resulted in strong economic vitality and business opportunities in Hainan Island, driving marked local economic development. The unique tropical and subtropical climate of Hainan Island, as well as its geographical location surrounded by the sea, provides children with superior conditions for outdoor activities and nurtures distinctive lifestyles and eating habits. However, relatively few studies have been conducted on overweight and obesity in preschool children, with most focusing on school-age children and adolescents. Particularly in Hainan, there is a lack of comprehensive surveys reflecting the overweight and obesity status of preschool children. Therefore, this study relied on the major science and technology program project of Hainan Island, “Study on the Monitoring of Children's Growth and Development and Influencing Factors in Hainan Island,” to comprehensively analyze the current situation of overweight and obesity among preschool children and their influencing factors in Hainan Island, which are influenced by the unique geographic environment and lifestyle and dietary habits, in the context of the free-trade port policy. This study aimed to fill the gap in epidemiological data on overweight and obesity among preschool children in tropical and subtropical regions of China. While differences in body mass and composition are well-documented across adult ethnic groups, these differences are also significant among children of the same age, sex, and ethnicity (11). Current domestic and international screening standards for childhood overweight and obesity, though not yet unified, predominantly rely on body mass index (BMI) percentile values based on age and sex (12). Therefore, this study utilized two internationally recognized standards and one local Chinese standard to comprehensively assess the prevalence of overweight and obesity among preschool children in Hainan Island. By analyzing the current situation and identifying influencing factors, this study will provide a scientific basis for the prevention and management of childhood overweight and obesity in Hainan Island and throughout China. In addition, the results will help optimize the development environment of the free trade port and promote the healthy growth of preschool children.

## 2 Materials and methods

### 2.1 Study design

This study was a cross-sectional survey conducted from January 2021 to June 2022 in Hainan Island, the largest free trade zone in China, and was reviewed and approved by the Ethics Committee of the Hainan Women's and Children's Medical Centre (No. 2021005). All parents or guardians of the participating children read and signed an informed consent form before participation.

Subjects were selected by multistage stratified random cluster sampling. The sample size was calculated using the following equations (10):

$$n=u_{\alpha /2}^{2}\pi (1-\pi )/\delta^{2}$$

$$n^{\prime }=n\times \mathrm{deff}=n\times [1+(n_{j}-1)\times \rho ]$$

where *n* is the sample size of simple randomized sampling; *α* is the inspection level, *α* = 0.05; *u* is the boundary value of the standard normal distribution corresponding to *α*, *u_α/2_* = 1.96; *π* is the estimated prevalence of overweight and obesity (13), *π* = 0.107; and *δ* is the allowable error, *δ* = 0.011. deff is the design effect; *n_j_* is the average group size, *n_j_* = 169; and *ρ* is the correlation coefficient within the group, *ρ* = 0.01; therefore, *n‘* = 8,131.12.We estimated a rejection rate of 10%; thus, the required sample size was 9,035 children, considering that some children may refuse to participate or may not complete the questionnaires. The number of clusters is the total number of samples divided by the group size, that is, 9,035/169 = 53.46.

### 2.2 Participants

This study employed a multistage stratified random cluster sampling method. In the first stage, based on a survey of under-five mortalities in Hainan Island, we divided the 18 cities and counties in Hainan Island into four types of areas. Type I areas included Haikou City and Sanya City; Type II areas included Qionghai City and Wenchang City; Type III areas included Wanning City, Ding'an County, Tunchang County, Chengmai County, Lingao County, Danzhou City, and Yangpu; and Type IV areas included Lingshui County, Ledong County, Baoting County, Wuzhishan City, Qiongzhong County, Dongfang City, Changjiang County, and Baisha County. In the second stage, 2–3 townships/streets were randomly selected in each city and county category, and each health hospital or community health service center in the selected townships or streets was used as a sampling point to include study participants who met the criteria.

The inclusion criteria for this study were as follows: (1) children aged 3–6 years and (2) children with household registration who have lived in Hainan Island for a long time or those who have moved from abroad but have lived in Hainan Island for more than two-thirds of their age. The exclusion criteria were as follows: (1) children with major diseases that seriously affected their growth and development, such as obvious physical disability or paralysis, and (2) children who did not cooperate with the survey and whose questionnaires were invalid.

### 2.3 Data collection and quality control

The questionnaire was designed to collect detailed data on children and their parents or guardians regarding the potential risk factors for overweight or obesity in children. We investigated the children's sex, age, place of residence, conception and delivery (including mode of conception, number of fetuses delivered, mode of delivery, disease at birth, gestational week of birth, and birth weight), family environment (type of family, main breadwinner, and whether they were an only child), dietary habits (habits of staple food texture and breakfast consumption), and lifestyle (daily outdoor exercise time, daily screen exposure time, and daily sleep time). For the children's parents or guardians, we examined personal and household characteristics such as height, weight, education level, and annual household income.

Each health or community health service center appointed 1–2 individuals in charge of the survey, with the person in charge being a physician from each health center or community health service center. Before the commencement of the project, special training was provided to the investigators, who were only allowed to conduct on-site investigations after passing the examination. On-site surveys were conducted face-to-face, with uniformly trained surveyors instructing the primary caregivers of the children to complete the survey. The questionnaires were reviewed by a designated quality inspector and members of the project team at various levels before collection; unqualified questionnaires were excluded. Questionnaire data were collected using the “Hainan Island Infant and Toddler Growth and Development Monitoring and Influencing Factors Research System” (now renamed as “Hainan Island Birth Cohort System,” Software Copyright Registration No. 10456369, website: https://dl.hnwcmc.com/#/login), using a two-person entry mode. Data inconsistencies were corrected by checking the original questionnaire before entering into the system.

### 2.4 Anthropometric measurements

Trained physicians performed standardized body measurements using standardized tools and methods. The examination included height and weight measurements using a standardized vertical electronic weight/height measuring device (model: vertical electronic weight/height measuring device, manufacturer: Seca, Germany; model: Seca255, measuring range: 20–205 cm, calibrated scale value: 1 mm).

#### 2.4.1 Weight measurement

The weights of the participants were measured using the weight-measuring instrument described above, and it was recommended to avoid taking measurements immediately after meals or exercise. Before taking the measurement, the participant was required to defecate and urinate; remove shoes, socks, hats, and outer clothing; and wear only a vest and shorts. Each child was instructed to stand at the center of the scale and keep their body steady without shaking or touching other objects. When the display readings were stable, they were recorded to two decimal places.

#### 2.4.2 Height measurement

Participants’ heights were measured using the aforementioned instrument. During the measurement, the participant, wearing only a vest and shorts, stood on the pedal in an upright position, chest out, abdomen in, arms naturally hanging down, knees together and straight, eyes looking straight ahead, and head in an upright position. The heel, hip, and angle between the two shoulder blades were required to touch the column simultaneously. The hand was used to hold the sliding board downward until its bottom surface and the top of the participant's head made contact. It was then confirmed whether the posture of the person being assessed was correct. If the posture was correct, the eyes of the participant and the bottom surface of the sliding board were aligned at the same level. The measurement was read where the bottom surface of the sliding board corresponded to the column value shown in “cm,” accurate to 0.1 cm. The physical examination equipment was used according to the manufacturer's specifications, and the equipment was calibrated regularly to ensure accuracy.

### 2.5 Definitions

#### 2.5.1 Baseline characteristics

Children's place of residence was divided into urban and rural areas according to China's “Statistical Urban and Rural Classification Code.” The conception method included natural conception or assisted reproductive techniques. Deliveries were classified as either normal or cesarean sections. The item on disease at birth examined whether the child was born with a neonatal illness. In terms of lifestyle habits, the child's daily screen time (the amount of time spent using electronic devices each day) and daily sleep time (the total sleep time at night and during the day) were assessed.

Regarding eating habits, children were assessed to determine whether they consumed breakfast regularly on a daily basis. The texture of staple food, ranging between paste, thin porridge, thick porridge, and soft or dry rice, was also examined. In terms of meal patterns, we noted unfavorable habits, such as feeding by a foster carer, chasing, and eating while watching TV or playing games, which were categorized as non-initiated eating behaviors. Fully active eating refers to the ability of children to independently and voluntarily engage in all aspects of the eating process, including the selection, intake, and mastication of food. This behavior is characterized by an active appetite and a proactive approach to eating, without the need for coercion or excessive guidance from parents or caregivers.

In terms of sociodemographic characteristics, types of families were categorized as follows: nuclear families, where children live with their parents; primary families, where children live with their grandparents; and other types of families. The family's economic level was classified into three bands (RMB per year) as follows: <30,000, 30,000–100,000, and >100,000. Additionally, we noted whether the primary caregiver was a parent, grandparent, or another individual. The educational level of parents was categorized as primary school and below, junior high school, secondary/high school, and college/undergraduate/graduate and above. In addition, the parents’ BMI was calculated based on self-reported height and weight.

#### 2.5.2 Overweight and obesity

In this study, childhood overweight and obesity were defined according to the WHO, Chinese, and International Obesity Task Force (IOTF) criteria.

According to the WHO guidelines, children aged 24–60 months are considered overweight or obese if their BMI-for-age z-score falls between 2 and 3 standard deviations (SD) or exceeds 3 SD, respectively (14). For children aged 61–83 months, overweight or obesity was defined as having a BMI-for-age z-score between 1 and 2 SD or >2 SD, respectively (15). Under the Chinese criteria, a BMI between 1 and 2 SD or more than 2 and 3 SD was defined as overweight or obesity, respectively, for children aged 0–84 months (16). The IOTF criteria for overweight and obesity are age- and sex-specific and are based on percentile curves passing through BMI cut-off points for overweight (25 kg/m^2^) and obesity (30 kg/m^2^) at 18 years of age (17).

### 2.6 Statistical analyses

All children enrolled in this study were categorized into two groups, namely the non-overweight and overweight/obesity groups, based on three distinct growth criteria utilized for defining childhood overweight and obesity. The distribution of continuous variables was assessed for normality using the Kolmogorov–Smirnov test. Non-normally distributed continuous variables are represented as medians and interquartile ranges, whereas normally distributed variables are presented as means and standard deviations. Categorical variables are presented as frequency counts and corresponding percentages. Baseline characteristics were compared between the groups using appropriate statistical tests, including the chi-squared test, *t*-test, or rank-sum test, as applicable. The prevalence of overweight and obesity among preschool children in Hainan Island was calculated using population-weighted analysis.

To identify statistically significant risk factors for overweight or obesity in childhood, univariate logistic regression analyses were first performed without any confounding factors. Then, Model 1 was adjusted for child sex, age, and region, as well as the mode of conception, gestational week of delivery, mode of delivery, number of fetuses delivered, birth weight, and disease at birth. Model 2 included the demographic characteristics such as the child's living and eating habits, household type, household income, primary caregiver, and parents, in addition to Model 1. The final risk factor was significantly and independently associated with the risk of being overweight or obese in preschool children, while significance persisted across three different growth criteria. The adjusted models were assessed for goodness-of-fit using the Hosmer–Lemeshow test to examine the consistency between the model predictions and the actual observed data. Statistical analysis was conducted using R version 4.3.1, with a predetermined alpha level of 0.05 indicating statistical significance.

## 3 Results

### 3.1 Baseline characteristics

The baseline characteristics of the children were stratified according to the Chinese, WHO, and IOTF criteria, as shown in Table 1. A total of 10,060 eligible preschool children with an average age of 4.89 years were enrolled in this study. The participants included 5,200 boys (51.7%) and 4,860 girls (48.3%), with 6,779 (67.4%) children from rural areas and 3,281 (32.6%) children from urban areas. The fathers of the majority of the participants [3,427 (34.1%)] had lower secondary education. The mothers of the majority of participants [3,969 (39.5%)] had junior high school education. In addition, the majority of the participants [7,324 (72.8%)] had parents as the main breadwinners. Regarding annual household income, the largest group consisted of 4,032 (40.1%) families with an annual income of less than RMB30,000.

**Table 1:** The baseline characteristics of the non-overweight and the overweight and obesity groups under three different growth criteria.

		Chinese criteria			WHO criteria			IOTF criteria		
		Non-overweight (*N* = 8,855)	Overweight/obesity (*N* = 1,205)	*P*	Non-overweight (*N* = 9,323)	Overweight/obesity (*N* = 737)	*P*	Non-overweight (*N* = 9,424)	Overweight/Obesity (*N* = 636)	*P*
Sex				1.000			<0.001			<0.001
Boy		4,577 (51.7%)	623 (51.7%)		4,716 (50.6%)	484 (65.7%)		4,716 (50.6%)	484 (65.7%)	
Girl		4,278 (48.3%)	582 (48.3%)		4,607 (49.4%)	253 (34.3%)		4,607 (49.4%)	253 (34.3%)	
Age				<0.001			<0.001			<0.001
3∼		1,434 (16.2%)	246 (20.4%)		1,652 (17.7%)	28 (3.8%)		1,652 (17.7%)	28 (3.8%)	
4∼		1,844 (20.8%)	241 (20%)		2,040 (21.9%)	45 (6.1%)		2,040 (21.9%)	45 (6.1%)	
5∼		1,789 (20.2%)	193 (16%)		1,823 (19.6%)	159 (21.6%)		1,823 (19.6%)	159 (21.6%)	
6∼		3,788 (42.8%)	525 (43.6%)		3,808 (40.8%)	505 (68.5%)		3,808 (40.8%)	505 (68.5%)	
Home address				<0.001			<0.001			<0.001
Urban		2,816 (31.8%)	465 (38.6%)		2,981 (32%)	300 (40.7%)		2,981 (32%)	300 (40.7%)	
Rural		6,039 (68.2%)	740 (61.4%)		6,342 (68%)	437 (59.3%)		6,342 (68%)	437 (59.3%)	
Father's BMI				<0.001			<0.001			<0.001
<28		6,453 (90.7%)	840 (86.4%)		6,806 (90.6%)	487 (85.3%)		6,806 (90.6%)	487 (85.3%)	
≥28		661 (9.3%)	132 (13.6%)		709 (9.4%)	84 (14.7%)		719 (9.5%)	74 (14.8%)	
Mother's BMI				<0.001			<0.001			<0.001
<28		7,378 (97.2%)	1,003 (94.4%)		7,797 (97.1%)	584 (93.7%)		7,867 (97.1%)	514 (93.6%)	
≥28		215 (2.8%)	59 (5.6%)		235 (2.9%)	39 (6.3%)		239 (2.9%)	35 (6.4%)	
Father's level of education				0.001			<0.001			0.010
Primary and below		143 (2%)	20 (2%)		145 (1.9%)	18 (3.1%)		150 (2%)	13 (2.6%)	
junior		3,063 (43.3%)	364 (37.2%)		3,226 (43.2%)	201 (35.1%)		3,247 (43%)	180 (35.8%)	
Secondary/High School		1,832 (25.9%)	260 (26.6%)		1,923 (25.7%)	169 (29.5%)		1,954 (25.9%)	138 (27.4%)	
College/Undergraduate/Postgraduate and above		2,033 (28.8%)	334 (34.2%)		2,182 (29.2%)	185 (32.3%)		2,195 (29.1%)	172 (34.2%)	
Mother's level of education				<0.001			0.059			0.003
Primary and below		203 (2.7%)	30 (2.8%)		210 (2.6%)	23 (3.7%)		211 (2.6%)	22 (4%)	
Junior		3,543 (47.2%)	426 (40.2%)		3,708 (46.7%)	261 (42.1%)		3,748 (46.8%)	221 (40.1%)	
Secondary/High School		1,768 (23.6%)	253 (23.9%)		1,874 (23.6%)	147 (23.7%)		1,892 (23.6%)	129 (23.4%)	
College/Undergraduate/Postgraduate and above		1,990 (26.5%)	351 (33.1%)		2,152 (27.1%)	189 (30.5%)		2,162 (27%)	179 (32.5%)	
Father's occupation				<0.001			<0.001			<0.001
Agriculture		1,718 (23.8%)	272 (27.6%)		1,808 (23.7%)	182 (31.6%)		1,837 (23.9%)	153 (30.2%)	
Business/services		1,764 (24.5%)	286 (29%)		1,892 (24.8%)	158 (27.4%)		1,911 (24.9%)	139 (27.5%)	
Specialist/civil servant/worker		2,642 (36.6%)	242 (24.6%)		2,763 (36.3%)	121 (21%)		2,770 (36%)	114 (22.5%)	
unemployed		394 (5.5%)	67 (6.8%)		419 (5.5%)	42 (7.3%)		423 (5.5%)	38 (7.5%)	
Serviceman and others		693 (9.6%)	118 (12%)		738 (9.7%)	73 (12.7%)		749 (9.7%)	62 (12.3%)	
Mother's occupation			<0.001			<0.001				<0.001
Agriculture		1,775 (23.7%)	280 (26.5%)		1,877 (23.7%)	178 (28.6%)		1,909 (23.8%)	146 (26.7%)	
Business/services		1,488 (19.9%)	253 (24%)		1,606 (20.3%)	135 (21.7%)		1,616 (20.2%)	125 (22.9%)	
Specialist/civil servant/worker		2,678 (35.7%)	251 (23.8%)		2,799 (35.3%)	130 (20.9%)		2,805 (35%)	124 (22.7%)	
unemployed		872 (11.6%)	153 (14.5%)		928 (11.7%)	97 (15.6%)		943 (11.8%)	82 (15%)	
Servicewoman and others		683 (9.1%)	119 (11.3%)		719 (9.1%)	83 (13.3%)		733 (9.2%)	69 (12.6%)	
Method of conception for a child				0.354			0.719			1.000
Natural conception		7,653 (97.9%)	1,076 (98.4%)		8,102 (98%)	627 (97.7%)		8,173 (97.9%)	556 (97.9%)	
Assisted reproduction		166 (2.1%)	18 (1.6%)		169 (2%)	15 (2.3%)		172 (2.1%)	12 (2.1%)	
Multiple pregnancy				0.017			0.068			0.024
No		7,229 (94%)	1,042 (95.9%)		7,658 (94.1%)	613 (95.9%)		7,725 (94.1%)	546 (96.5%)	
Yes		461 (6%)	45 (4.1%)		480 (5.9%)	26 (4.1%)		486 (5.9%)	20 (3.5%)	
Mode of delivery				<0.001			0.089			0.304
Vaginal delivery		7,666 (86.7%)	979 (81.3%)		7,995 (85.9%)	650 (88.2%)		8,107 (86.1%)	538 (84.6%)	
Cesarean section		1,178 (13.3%)	225 (18.7%)		1,316 (14.1%)	87 (11.8%)		1,305 (13.9%)	98 (15.4%)	
Congenital disease				0.979			0.690			1.000
No		7,596 (97.6%)	1,062 (97.7%)		8,036 (97.6%)	622 (98%)		8,108 (97.6%)	550 (97.7%)	
Yes		184 (2.4%)	25 (2.3%)		196 (2.4%)	13 (2%)		196 (2.4%)	13 (2.3%)	
Gestational age at birth		39.00[38.00,40.00]	39.00[38.00,40.00]	0.006	39.00[38.00,40.00]	39.00[38.00,40.00]	0.048	39.00[38.00,40.00]	39.00[38.00,40.00]	0.282
Birth weight of the child		3.10[2.89,3.40]	3.30[3.00,3.56]	0.000	3.10[2.90,3.40]	3.30[3.00,3.50]	0.000	3.10[2.90,3.40]	3.30[3.00,3.52]	0.000
Type of family				0.074			0.008			0.041
Nuclear families		4,964 (63.5%)	731 (66.8%)		5,249 (63.5%)	446 (69.5%)		5,305 (63.6%)	390 (68.7%)	
Primary families		2,212 (28.3%)	288 (26.3%)		2,351 (28.4%)	149 (23.2%)		2,358 (28.3%)	142 (25%)	
Others		643 (8.2%)	75 (6.9%)		671 (8.1%)	47 (7.3%)		682 (8.2%)	36 (6.3%)	
Primary caregiver				0.072			<0.001			0.023
Parent		6,405 (81.9%)	919 (84.1%)		6,763 (81.8%)	561 (87.5%)		6,838 (82%)	486 (85.7%)	
Grandparent		1,285 (16.4%)	152 (13.9%)		1,373 (16.6%)	64 (10%)		1,368 (16.4%)	69 (12.2%)	
Another individual		126 (1.6%)	22 (2%)		132 (1.6%)	16 (2.5%)		136 (1.6%)	12 (2.1%)	
Single-child family				0.012			0.006			0.012
No		5,725 (73.2%)	761 (69.6%)		6,049 (73.1%)	437 (68.1%)		6,099 (73.1%)	387 (68.1%)	
Yes		2,094 (26.8%)	333 (30.4%)		2,222 (26.9%)	205 (31.9%)		2,246 (26.9%)	181 (31.9%)	
Annual household income				<0.001			<0.001			<0.001
<30,000		3,609 (48.2%)	423 (39.4%)		3,776 (47.6%)	256 (40.3%)		3,809 (47.6%)	223 (39.8%)	
≥30,000, ≤100,000		2,901 (38.7%)	463 (43.2%)		3,105 (39.2%)	259 (40.8%)		3,131 (39.1%)	233 (41.6%)	
>100,000		983 (13.1%)	187 (17.4%)		1,050 (13.2%)	120 (18.9%)		1,066 (13.3%)	104 (18.6%)	
Texture of staple food				0.004			0.012			0.003
Paste, thin porridge, thick Porridge		3,007 (38.5%)	371 (33.9%)		3,165 (38.3%)	213 (33.2%)		3,197 (38.3%)	181 (31.9%)	
Soft or dry rice		4,811 (61.5%)	723 (66.1%)		5,105 (61.7%)	429 (66.8%)		5,147 (61.7%)	387 (68.1%)	
Breakfast habits				0.022			0.024			0.107
No		191 (2.4%)	14 (1.3%)		199 (2.4%)	6 (0.9%)		198 (2.4%)	7 (1.2%)	
Yes		7,628 (97.6%)	1,080 (98.7%)		8,072 (97.6%)	636 (99.1%)		8,147 (97.6%)	561 (98.8%)	
Eating behaviour				<0.001			<0.001			<0.001
Non-initiated eating behaviors		3,286 (42%)	342 (31.3%)		3,477 (42%)	151 (23.5%)		3,479 (41.7%)	149 (26.2%)	
Fully active eating		4,532 (58%)	752 (68.7%)		4,793 (58%)	491 (76.5%)		4,865 (58.3%)	419 (73.8%)	
Outdoor exercise time		2.00[1.00,3.00]	2.00[1.00,3.00]	0.624	2.00[1.00,3.00]	2.00[1.00,3.00]	0.001	2.00[1.00,3.00]	2.00[1.00,3.00]	0.066
Exposure to screens		1.00[1.00,2.00]	1.00[1.00,2.00]	0.579	1.00[1.00,3.00]	2.00[1.00,3.00]	0.967	1.00[1.00,2.00]	1.00[1.00,2.00]	0.604
Average sleep duration		10.82[10.20,11.44]	10.72[10.00,11.35]	0.916	10.82[10.20,11.44]	10.72[10.00,11.35]	0.002	10.82[10.19,11.44]	10.71[10.01,11.38]	0.006

### 3.2 Prevalence of overweight and obesity among 3–6-year-old children in Hainan Island

**Figure 1:**
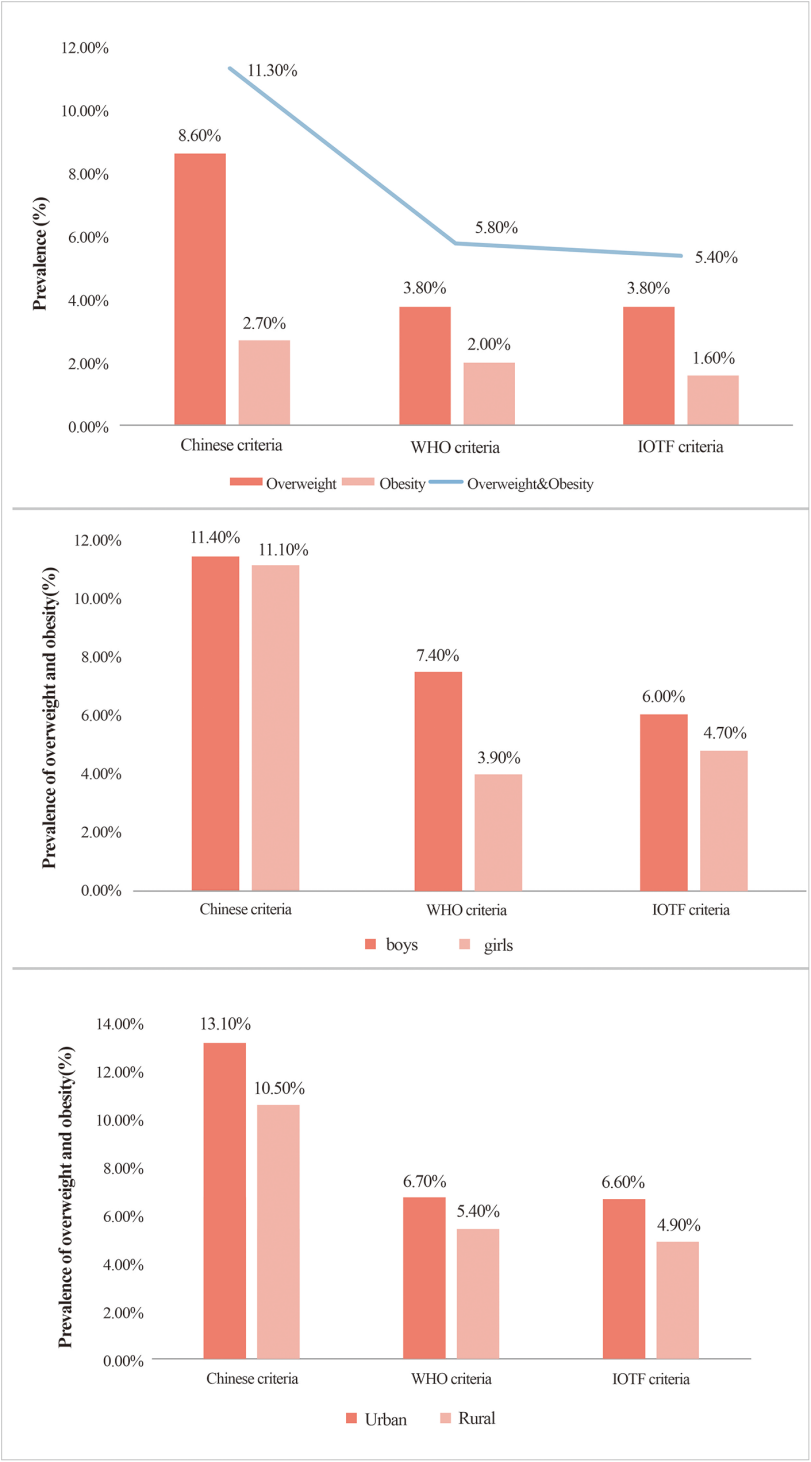
Prevalence of overweight and obesity among preschool children in Hainan Island: an analysis based on different criteria, gender, and urban-rural distribution..

The prevalence of overweight and obesity estimated using the Chinese criteria (11.30%) was significantly higher than those estimated using the WHO (5.80%) and IOTF (5.40%) criteria (*χ*^2^ = 12,870.368, *P* *<* 0.001). The prevalence of overweight and obesity among boys tended to be higher than that among girls when the three assessment standards were used (*P* < 0.05). Simultaneously, the prevalence of overweight and obesity among children in urban areas was generally higher than that among children in rural areas, regardless of the criteria (*P* < 0.001) (Figure 1).

### 3.3 Identification of significant risk factors

Given that sex, age, and regional area are unmodifiable demographic factors, we deemed them confounding variables. The other significant factors were examined using logistic regression analyses to build the final models. Parental occupation, parental obesity, only child status, main breadwinner, family income, eating habits, texture of staple food, and birth weight were significantly associated with the risk of being overweight or obese in children at the 5% significance level according to the three growth criteria (Table 2). After multivariable adjustment, fathers with obesity [odds ratio [OR]: 1.69, 1.86, 1.88; 95% confidence interval [CI]: 1.35–2.12, 1.40–2.46, 1.41–2.51; *P* < 0.001, *P* < 0.001, *P* < 0.001; respectively, according to Chinese, WHO, and IOTF criteria], mothers with obesity (OR: 1.76, 1.78, 1.86; 95% CI: 1.22–2.54, 1.13–2.80, 1.18–2.93; *P* = 0.002, *P* = 0.013, *P* = 0.008; respectively, according to Chinese, WHO, and IOTF criteria), a household income >RMB100,000 (OR: 1.39, 1.69, 1.59; 95% CI: 1.08–1.79, 1.23–2.32, 1.14–2.22; *P* = 0.011, *P* = 0.001, *P* = 0.007; respectively, according to Chinese, WHO, and IOTF criteria), fully active eating (OR: 1.75, 1.62, 1.66; 95% CI: 1.48–2.08, 1.28–2.03, 1.31–2.10; *P* < 0.001, *P* < 0.001, *P* < 0.001; respectively, according to Chinese, WHO, and IOTF criteria), and birth weight (OR: 2.21, 1.97, 1.91; 95% CI: 1.84–2.65, 1.57–2.49, 1.51–2.42; *P* < 0.001, *P* < 0.001, *P* < 0.001; respectively, according to Chinese, WHO, and IOTF criteria) were consistently and significantly associated with childhood overweight and obesity according to the three growth criteria at the 5% significance level (Table 3).

**Table 2:** Identification of significant contributing factors separately for overweight and obesity under three different criteria using univariate logistic regression analyses in preschool-aged children.

			Chinese criteria			WHO criteria			IOTF criteria	
		OR	95%CI	*P*	OR	95%CI	*P*	OR	95%CI	*P*
Sex										
Boy			Ref			Ref			Ref	
Girl		1.00	0.89–1.13	0.993	0.54	0.46–0.63	<0.001	0.76	0.64–0.89	<0.001
Age										
3∼			Ref			Ref			Ref	
4∼		0.76	0.63–0.92	0.005	1.30	0.81–2.10	0.278	1.51	1.09–2.10	0.014
5∼		0.63	0.51–0.77	<0.001	5.15	3.43–7.73	<0.001	1.72	1.24–2.38	0.001
6∼		0.81	0.69–0.95	0.010	7.82	5.33–11.50	<0.001	2.60	1.96–3.46	<0.001
Home address										
Urban			Ref			Ref			Ref	
Rural		0.74	0.66–0.84	<0.001	0.68	0.59–0.80	<0.001	0.65	0.55–0.77	<0.001
Father's BMI										
<28			Ref			Ref			Ref	
≥28		1.53	1.26–1.87	<0.001	1.66	1.30–2.11	<0.001	1.66	1.28–2.15	<0.001
Mother's BMI										
<28			Ref			Ref			Ref	
≥28		2.02	1.50–2.71	<0.001	2.22	1.56–3.14	<0.001	2.24	1.55–3.23	<0.001
Father's level of education										
Primary and below			Ref			Ref			Ref	
Junior		0.85	0.53–1.37	0.506	0.50	0.30–0.84	0.008	0.64	0.36–1.15	0.135
Secondary/High School		1.01	0.62–1.65	0.953	0.71	0.42–1.18	0.188	0.81	0.45–1.47	0.498
College/Undergraduate/Postgraduate and above		1.17	0.73–1.90	0.513	0.68	0.41–1.14	0.145	0.90	0.50–1.63	0.737
Mother's level of education										
Primary and below			Ref			Ref			Ref	
Junior		0.81	0.55–1.21	0.308	0.64	0.41–1.01	0.053	0.57	0.36–0.90	0.015
Secondary/High School		0.97	0.65–1.45	0.876	0.72	0.45–1.14	0.157	0.65	0.41–1.05	0.079
College/Undergraduate/Postgraduate and above		1.19	0.80–1.78	0.386	0.80	0.51–1.26	0.342	0.79	0.50–1.26	0.331
Father's occupation										
Agriculture			Ref			Ref			Ref	
Business/services		1.02	0.86–1.22	0.794	0.83	0.66–1.04	0.100	0.87	0.69–1.11	0.266
Specialist/civil servant/worker		0.58	0.48–0.70	<0.001	0.44	0.34–0.55	<0.001	0.49	0.38–0.63	<0.001
Unemployed		1.07	0.80–1.43	0.628	1.00	0.70–1.42	0.981	1.08	0.74–1.56	0.689
Serviceman and others		1.08	0.85–1.36	0.541	0.98	0.74–1.31	0.904	0.99	0.73–1.35	0.969
Mother's occupation										
Agriculture			Ref			Ref			Ref	
Business/services		1.08	0.90–1.29	0.423	0.89	0.70–1.12	0.311	1.01	0.79–1.30	0.929
Specialist/civil servant/worker		0.59	0.50–0.71	<0.001	0.49	0.39–0.62	<0.001	0.58	0.45–0.74	<0.001
Unemployed		1.11	0.90–1.38	0.328	1.10	0.85–1.43	0.462	1.14	0.86–1.51	0.371
Servicewoman and others		1.10	0.88–1.39	0.401	1.22	0.93–1.60	0.160	1.23	0.91–1.66	0.173
Method of conception for a child										
Natural conception			Ref			Ref			Ref	
Assisted reproduction		0.77	0.47–1.26	0.299	1.15	0.67–1.96	0.615	1.03	0.57–1.85	0.933
Multiple pregnancy										
No			Ref			Ref			Ref	
Yes		0.68	0.50–0.93	0.015	0.68	0.45–1.01	0.058	0.58	0.37–0.92	0.020
Mode of delivery										
Vaginal delivery			Ref			Ref			Ref	
Cesarean section		1.50	1.28–1.75	<0.001	0.81	0.65–1.02	0.080	1.13	0.91–1.41	0.277
Congenital disease										
No			Ref			Ref			Ref	
Yes		0.97	0.64–1.48	0.894	0.86	0.49–1.51	0.594	0.98	0.55–1.73	0.938
Gestational age at birth		1.05	1.01–1.09	0.018	1.05	1.00–1.10	0.077	1.02	0.97–1.08	0.390
birth weight of the child		2.17	1.90–2.48	<0.001	2.05	1.75–2.41	<0.001	1.94	1.64–2.31	<0.001
type of family										
Nuclear families			Ref			Ref			Ref	
Primary families		0.88	0.76–1.02	0.097	0.75	0.62–0.90	0.003	0.82	0.67–1.00	0.048
Others		0.79	0.62–1.02	0.069	0.82	0.60–1.13	0.224	0.72	0.51–1.02	0.064
Primary caregiver										
Parent			Ref			Ref			Ref	
Grandparent		0.82	0.69–0.99	0.037	0.56	0.43–0.73	<0.001	0.71	0.55–0.92	0.009
Another individual		1.22	0.77–1.92,	0.401	1.46	0.86–2.47	0.158	1.24	0.68–2.26	0.478
Single-child family										
No			Ref			Ref			Ref	
Yes		1.20	1.04–1.37	0.011	1.28	1.07–1.52	00.006	1.27	1.06–1.53	0.010
Annual household income										
<30,000			Ref			Ref			Ref	
≥30,000, ≤100,000		1.36	1.18–1.57	<0.001	0.75	0.62–0.90	0.003	0.82	0.67–1.00	0.048
>100,000		1.62	1.35–1.95	<0.001	0.82	0.60–1.13	0.224	0.72	0.51–1.02	0.064
Texture of staple food										
Paste, thin porridge, thick porridge			Ref			Ref			Ref	
Soft or dry rice		1.22	1.07–1.39	0.004	1.25	1.05–1.48	0.010	1.33	1.11–1.59	0.002
Breakfast habits										
No			Ref			Ref			Ref	
Yes		1.93	1.12–3.34	0.018	2.61	1.16–5.91	0.021	1.95	0.91–4.16	0.085
Eating behaviour										
Non-initiated eating behaviors			Ref			Ref			Ref	
Fully active eating		1.59	1.39–1.83	<0.001	2.36	1.96–2.85	<0.001	1.33	1.11–1.59	0.002
Outdoor exercise time		0.99	0.95–1.02	0.487	0.95	0.90–0.99	0.026	0.97	0.93–1.02	0.283
Exposure to screens		1.02	0.97–1.07	0.438	1.00	0.94–1.07	0.970	1.02	0.95–1.09	0.583
Average sleep duration		1.00	0.93–1.06	0.890	0.89	0.82–0.96	0.005	0.90	0.82–0.98	0.013

**Table 3:** Identification of significant contributing factors separately for overweight and obesity under three different criteria using multivariable logistic regression analyses in preschool-aged children.

		Chinese criteria						WHO criteria						IOTF criteria					
		Model 1			Model 2			Model 1			Model 2			Model 1			Model 2		
		OR	95%CI	*P*	OR	95%CI	*P*	OR	95%CI	*P*	OR	95%CI	*P*	OR	95%CI	*P*	OR	95%CI	*P*
Father's occupation																			
Agriculture			Ref			Ref			Ref			Ref			Ref			Ref	
Business/services		1.03	0.86–1.24	0.719	0.98	0.78–1.22	0,837	0.95	0.75–1.20	0.649	0.90	0.68–1.18	0.440	0.92	0.72–1.18	0.528	0.93	0.69–1.25	0.623
Specialist/civil servant/worker		0.65	0.53–0.79	<0.001	0.74	0.54–1.01	0.057	0.48	0.37–0.62	<0.001	0.53	0.35–0.80	0.002	0.56	0.43–0.73	<0.001	0.66	0.43–1.02	0.062
unemployed		1.07	0.80–1.45	0.634	1.04	0.73–1.48	0.832	0.76	0.53–1.10	0.142	0.87	0.56–1.34	0.527	0.95	0.65–1.39	0.802	1.16	0.74–1.83	0.520
Serviceman and others		1.07	0.85–1.37	0.558	1.10	0.82–1.48	0.511	0.87	0.65–1.17	0.366	0.92	0.65–1.32	0.668	0.93	0.68–1.27	0.640	1.07	0.73–1.56	0.737
Mother's occupation																			
Agriculture			Ref			Ref			Ref			Ref			Ref			Ref	
Business/services		1.08	0.89–1.30	0.431	1.11	0.86–1.42	0.417	1.03	0.81–1.32	0.786	1.00	0.72–1.37	0.979	1.09	0.84–1.40	0.521	0.97	0.70–1.36	0.871
Specialist/civil servant/worker		0.66	0.54–0.80	<0.001	0.98	0.72–1.34	0.912	0.54	0.42–0.70	<0.001	1.02	0.68–1.54	0.916	0.66	0.51–0.85	0.002	1.03	0.67–1.58	0.905
unemployed		1.12	0.90–1.39	0.314	1.33	1.01–1.77	0.045	0.89	0.68–1.17	0.396	1.15	0.81–1.63	0.423	1.02	0.76–1.35	0.912	1.11	0.76–1.61	0.582
Servicewoman and others		1.11	0.88–1.41	0.376	1.18	0.87–1.60	0.284	1.01	0.76–1.35	0.921	1.16	0.80–1.67	0.430	1.10	0.81–1.49	0.548	1.15	0.78–1.70	0.479
Father's BMI																			
<28			Ref			Ref			Ref			Ref			Ref			Ref	
≥28		1.59	1.29–1.95	<0.001	1.69	1.35–2.12	<0.001	1.77	1.36–2.29	<0.001	1.86	1.40–2.46	<0.001	1.73	1.32–2.26	<0.001	1.88	1.41–2.51	<0.001
Mother's BMI																			
<28			Ref			Ref			Ref			Ref			Ref			Ref	
≥28		1.88	1.39–2.55	<0.001	1.76	1.22–2.54	0.002	1.99	1.37–2.90	<0.001	1.78	1.13–2.80	0.013	1.97	1.35–2.88	*p* < 0.001	1.86	1.18–2.93	0.008
Single-child family																			
No			Ref			Ref			Ref			Ref			Ref			Ref	
Yes		1.13	0.98–1.30	0.090	1.17	0.99–1.38	0.067	1.17	0.98–1.41	0.083	1.22	0.98–1.50	0.069	1.17	0.97–1.41	0.100	1.18	0.94–1.47	0.146
Primary caregiver																			
Parent			Ref			Ref			Ref			Ref			Ref			Ref	
Grandparent		0.88	0.73–1.07	0.205	1.01	0.80–1.28	0.925	0.90	0.68–1.19	0.451	1.05	0.75–1.49	0.761	0.95	0.72–1.24	0.695	1.06	0.76–1.47	0.751
Another individual		1.17	0.74–1.87	0.497	1.09	0.64–1.85	0.755	1.24	0.72–2.13	0.441	1.32	0.72–2.45	0.371	1.10	0.60–2.02	0.754	1.35	0.71–2.58	0.361
Annual household income																			
<30,000			Ref			Ref			Ref			Ref			Ref			Ref	
≥30,000, ≤100,000		1.26	1.09–1.46	0.002	1.25	1.04–1.50	0.017	1.25	1.03–1.51	0.021	1.17	0.92–1.47	0.196	1.21	1.00–1.48	0.053	1.24	0.97–1.58	0.089
>100,000		1.40	1.15–1.71	<0.001	1.39	1.08–1.79	0.011	1.86	1.45–2.39	<0.001	1.69	1.23–2.32	0.001	1.58	1.22–2.05	<0.001	1.59	1.14–2.22	0.007
Eating behaviour																			
Non-initiated eating behaviors			Ref			Ref			Ref			Ref			Ref			Ref	
Fully active eating		1.69	1.46–1.96	<0.001	1.75	1.48–2.08	<0.001	1.62	1.33–1.97	<0.001	1.62	1.28–2.03	<0.001	1.70	1.39–2.08	<0.001	1.66	1.31–2.10	<0.001
Texture of staple food																			
Paste, thin porridge, thick porridge			Ref			Ref			Ref			Ref			Ref			Ref	
soft or dry rice		1.13	0.98–1.30	0.088	1.02	0.86–1.20	0.836	1.29	1.08–1.55	0.005	1.05	0.85–1.30	0.635	1.28	1.06–1.55	0.010	1.11	0.89–1.39	0.357
Birth weight of the child		2.19	1.88–2.54	<0.001	2.21	1.84–2.65	<0.001	1.94	1.60–2.36	<0.001	1.97	1.57–2.49	<0.001	1.94	1.59–2.36	<0.001	1.91	1.51–2.42	<0.001
Hosmer-Lemeshow					X-squared	df	*P*				X-squared	df	*P*				X-squared	df	*P*
					6.17	8.00	0.628				6.36	8.00	0.606				11.42	8.00	0.179

Model 1: Adjusted for gender, age, region, parity, mode of delivery, conception method, gestational age at birth, birth weight, and birth-related diseases. Model 2: Adjusted for region, age, gender, parity, mode of delivery, conception method, gestational age at birth, birth weight, birth-related diseases, parental education level, parental occupation, types of families, primary breadwinner, screen time, outdoor activity, amount of time spent, economic level of the family, habit of consuming breakfast, texture of the staple food, and dining methods.

## 4 Discussion

To the best of our knowledge, this study is the first to use three different criteria for assessing overweight and obesity to investigate the prevalence of overweight and obesity in preschool children in Hainan Island, China's largest free trade zone, with the Chinese standard more accurately identifying overweight and obese preschool children. When applying these three assessment criteria among preschool children in Hainan Island, the prevalence of overweight and obesity was higher among boys than among girls, and urban children exhibited considerably higher rates of overweight and obesity than rural children.

We used the Chinese, WHO, and IOTF criteria to assess each child's weight status using BMI to determine whether they were overweight or obese. This method was chosen because BMI is the most widely used anthropometric indicator for assessing weight status (18). However, the interpretation of BMI in children and adolescents varies according to age and sex, while differences in the threshold values of BMI in adults may lead to the misclassification of overweight and obesity (10). In addition, there is no standardized definition of childhood obesity. A study covering several Asian countries, including China, Lebanon, Malaysia, the Philippines, and Thailand, showed that the use of a single BMI cutoff point may not be appropriate for health risk screening of all Asian children, as the relationship between BMI and percent body fat varies by ethnicity in Asian children (19). In view of this, we applied a combination of criteria in this study with the aim of more accurately assessing the prevalence of overweight and obesity among preschool children in Hainan Island.

The Chinese, WHO, and IOTF criteria were used to evaluate the prevalence of overweight and obesity among children aged 3–6 years in Hainan Island. The results showed that the prevalence of overweight and obesity estimated using the Chinese criteria was the highest compared to that estimated using the WHO and IOTF criteria. Similarly, in two studies on the prevalence of overweight and obesity in preschool children in Beijing and Hunan provinces of China, the prevalence of overweight and obesity in preschool children estimated using the Chinese criteria was found to be considerably higher than that using the WHO and IOTF criteria (10, 20). This difference may stem from the characteristics of the populations on which the different standards were developed. The Chinese standards are based on the growth and development of Chinese children, whereas the IOTF standards are based on data from individuals under 25 years of age in six regions: Brazil, the United Kingdom, Hong Kong (China), the Netherlands, Singapore, and the United States (16, 19). In contrast, the WHO criteria are based on a sample of children who exhibited optimal growth (21, 22). Owing to different reference populations, the definition of the threshold for childhood overweight and obesity varies for each criterion, which ultimately leads to different results when assessing the prevalence of overweight and obesity according to the three criteria.

Based on the preliminary findings of our research group, the reference values for height, weight, and head circumference of boys and girls younger than 7 years in Hainan Island are generally lower than the WHO standards, and this discrepancy tends to widen with increasing age (23). Considering that the growth and development levels of children under 7 years of age in Hainan Province are generally lower than the international average, the Chinese standard can more accurately identify preschool children who are overweight or obese in comparison with the two international standards. Therefore, when screening for overweight and obesity among preschool children in the Hainan Province, the use of Chinese standards may be more appropriate and accurate.

The prevalence of overweight and obesity among preschool children in Hainan was lower than that reported in other parts of the world. Compared with the results of previous studies using the WHO criteria, the prevalence of overweight and obesity among preschool children in Hainan Island was lower than the corresponding prevalence among preschool children in the Thessaloniki region of Greece (32.6%) (24), the Quebec region of Canada (26.3%) (25), and ten cities in India (23.9%) (26). Additionally, compared with studies that used the IOTF criteria, the prevalence of overweight and obesity among preschool children in Hainan Island was considerably lower than the corresponding prevalence among preschool children in the Thessaloniki region of Greece (21.2%) (24), the Quebec region of Canada (16.6%) (25), ten cities in India (18.2%) (26), and the northern region of Vietnam (overweight: 16.7%; obesity: 4.5%) (27). This may be because the development of overweight and obesity in children is influenced by a combination of individual, familial, community, and social factors (1). China, Vietnam, and India are developing countries, whereas Canada and Greece are developed countries with remarkable differences in economic and cultural aspects.

Compared to data from other regions of China, the prevalence of overweight and obesity among preschool children in Hainan Island was relatively low. Moreover, compared to studies using WHO standards, the prevalence of overweight and obesity among preschool children in Hainan Island was considerably lower than that in the six cities of Northeast China (overweight, 10.93%; obesity, 13.81%) (28), Beijing (15.12%) (20), and Hunan Province (overweight, 4.5%; obesity, 2.1%) (10). Similarly, according to the IOTF standards, the prevalence of overweight and obesity among preschool children in Hainan Island was lower than that reported in the 2011 China Health and Nutrition Survey (overweight, 10.1%; obesity, 12.4%) (28), the six cities of Northeast China (overweight, 10.98%; obesity, 6.08%), Beijing (15.58%) (20), and Hunan Province (overweight, 4.6%; obesity 2.0%) (10). Additionally, according to Chinese standards, the prevalence of overweight and obesity among preschool children in Hainan Island was lower than that in Beijing (22.47%), Changsha City (overweight, 15.2%; obesity, 9.8%) (29), and the overall prevalence in Hunan Province (overweight, 9.1%; obesity, 4.3%) (10). Thus, the findings of this study are consistent with those of previous studies. Over the past two decades, the prevalence of overweight and obesity in northern and northeastern China has been notably higher than that in coastal provinces such as Guangdong and Hainan, as well as in economically less developed regions such as Guangxi, Guizhou, and Qinghai (30–33). We infer that the prevalence of overweight and obesity among preschool children in Hainan Island remains relatively low compared to that in urban areas in other parts of China. This may be closely associated with the comparatively low level of economic development in Hainan Island and its unique geographical characteristics.

According to the three assessment criteria, the prevalence of both overweight and obesity among preschool children in the urban areas of Hainan Island was significantly higher than that in rural areas, a finding that is consistent with previous studies in China (4, 31). This difference may stem from the fact that children in urban areas are more likely to be exposed to high-calorie, high-fat foods, such as Western fast foods, fried foods, and sweetened beverages (34, 35). In addition, urban children in this age group may be more inclined to adopt sedentary lifestyles, such as watching television for long periods, indulging in video games, or attending training programs outside kindergarten (e.g., foreign languages, drawing, and piano) (36).

In this study, we observed that the prevalence of overweight and obesity among preschool boys in Hainan Island was higher than that among girls according to the Chinese, WHO, and IOTF standards, which is consistent with previous research findings in China (10, 20, 28, 37, 38). However, in most European countries, the prevalence of overweight and obesity is higher among preschool girls than among boys (39). This may be because Chinese girls are more concerned about the impact of food on their health and weight, whereas boys tend to consume snacks such as drinks and crisps and spend more time watching television and playing video games (40–42). Furthermore, Chinese families tend to idealize larger, more muscular male body shapes, and they are less inclined to encourage their sons to lose weight due to traditional sex preferences favoring boys (10, 43).

Another important finding of this study was the identification of five key risk factors, namely paternal obesity, maternal obesity, high family income, fully active eating, and high birth weight, independently associated with the risk of being overweight and obese among preschool children in Hainan Island according to the three criteria.

The finding that parental overweight and obesity are important risk factors for the development of overweight and obesity in children was consistently verified in our study and several previous studies (43–46). This may be because genetic and environmental factors play crucial roles in the development of overweight and obesity in children (47–49). Parental overweight or obesity may not only increase genetic susceptibility to childhood overweight and obesity but may also be a representative factor of environmental exposure that contributes to childhood obesity (46). Therefore, to address the prevalence of childhood overweight and obesity, the key factors of parental overweight and obesity must be considered.

This study's findings indicate that children from high-income families are at a comparatively higher risk of becoming overweight or obese. This may be because preschool children in high-income families are more frequently exposed to high-calorie, high-fat foods, such as Western fast foods, fried foods, and sweetened beverages (9). Simultaneously, with the increased use of electronic devices in high-income families, children's exercise time has reduced, and sedentary lifestyles have become more common. Poor dietary patterns and sedentary habits can lead to increased insulin resistance in children, which in turn increases the risk of being overweight and obese (1). Therefore, for high-income families, paying attention to their children's dietary health, arranging their work and rest schedules reasonably, and encouraging children to participate in more outdoor activities and sports are crucial for preventing and reducing the prevalence of overweight and obesity among children.

Furthermore, the results of our study show that increased birth weight increases the risk of being overweight and obese in children, a finding that is consistent with those of several previous studies (50–52). This may be because fetal growth and development *in utero* shapes the physiology and structure that affect the risk of childhood obesity, and birth weight is a marker of fetal growth and the prenatal environment (53, 54). In addition, a pleiotropic genetic effect between birth weight and childhood obesity has been demonstrated (54). Focusing on the effects of early-life traits will not only help us understand their complex genetic structure but also provide strong support for the development of effective early-life interventions to prevent the risk of overweight and obesity among children and promote healthy childhood development.

Finally, our findings showed that children with fully active eating habits had a relatively higher risk of being overweight and obese. This may be because these children usually have a stronger appetite and may be more inclined to eat high-calorie and high-fat foods when making food choices (55). Additionally, they often find it difficult to control the amount of food they eat. Therefore, to prevent and reduce the problems of overweight and obesity in children, parents should pay attention to and guide their children's eating behaviors, help them develop healthy eating habits, control their appetite appropriately, and make rational food choices.

To effectively address the issue of obesity in preschool children, comprehensive measures must be implemented to promote collaboration between society, families, and schools. First, special attention should be given to high-income families and children with parents who are obese. Targeted health education and behavioral interventions should be implemented to encourage these families to adopt healthier lifestyles. Personalized health management advice can be provided through family doctors, community health centers, and other channels, enabling early prevention and intervention for obesity. Second, for children who are fully in control of their eating, dietary behavior intervention programs should be carried out in schools or communities. These programs would help both parents and children understand the risks of overeating and obesity associated with unrestricted eating and encourage children to eat according to their actual needs rather than overindulging. Simultaneously, health education on proper nutrition should be promoted in schools to teach children how to make healthy food choices and control portion sizes, fostering healthy eating habits. Finally, the health of children with high birth weight should receive attention. Specialized health monitoring and management programs should be established, particularly in the early stages of child development. Regular weight monitoring and health assessments will help track the physical development of high-birth-weight children. Scientific dietary guidance and appropriate physical activity recommendations should be provided to assist parents in effectively managing their children's weight gain, thus reducing the risk of obesity.

This study employed a multistage stratified random cluster sampling method across the entire island, with a large and widely distributed sample size, ensuring the representativeness and broad applicability of the sample. Therefore, the findings more accurately reflect the prevalence of overweight and obesity among preschool children in Hainan Island.

Although this study has a clear advantage in sample selection, its cross-sectional nature means it is not possible to determine whether the observed association between overweight/obesity and certain factors is causal. In addition, there may have been recall bias during the data collection process, which could have affected the accuracy of the results. Therefore, future studies should adopt a longitudinal design to more accurately investigate the causes of overweight and obesity and implement measures to reduce the impact of recall bias on the study outcomes.

It is important to note that diet plays a considerable role in the problem of overweight and obesity in children. This study, as part of a major scientific and technological project in Hainan Island titled “Research on Children's Growth and Development Monitoring and Influencing Factors in Hainan Island,” did not specifically focus on the dietary behaviors of preschool children in Hainan. Furthermore, this study used children's BMI to determine overweight and obesity; however, BMI cannot distinguish between muscle and fat, and thus cannot accurately differentiate between overweight or obesity due to fat gain or muscle growth. Future studies should consider using percentiles to better reflect the relative position of children within their age and sex groups. These limitations represent a gap in the study's scope.

In conclusion, the results of this large-scale epidemiological survey showed that the prevalence of overweight and obesity among preschool children in Hainan Island was low compared to that in some regions reported in national and international literature. Compared with the WHO and IOTF standards, the Chinese standards may be more suitable for screening overweight and obesity in preschool children in Hainan Island. The implementation of targeted interventions, such as encouraging parents to maintain a healthy weight, ensuring that the birth weight of newborns is within the normal range, enhancing health literacy, fostering healthy eating environments, and paying more attention to children from higher-income families, is important to reduce the risk of overweight and obesity in children.

## Data Availability

The original contributions presented in the study are included in the article/Supplementary Material, further inquiries can be directed to the corresponding author.

## References

[1] Jebeile H Kelly AS O'Malley G Baur LA. Obesity in children and adolescents: epidemiology, causes, assessment, and management. Lancet Diabetes Endocrinol. (2022) 10:351–65. 10.1016/S2213-8587(22)00047-X 35248172 PMC9831747

[2] Lobstein T Jackson-Leach R Moodie ML Hall KD Gortmaker SL Swinburn BA Child and adolescent obesity: part of a bigger picture. Lancet. (2015) 385:2510–20. 10.1016/S0140-6736(14)61746-3 25703114 PMC4594797

[3] Subspecialty Group of Endocrinologic HAMD, Editorial Board CJOP, Subspecialty Group of Child Health Care TSOP, Subspecialty Group of Clinical Nutrition TSOP. Expert consensus on diagnosis, assessment, and management of obesity in chinese children. Zhonghua Er Ke Za Zhi. (2022) 60:507–15. 10.3760/cma.j.cn112140-20220112-00043 35658354

[4] Pan XF Wang L Pan A. Epidemiology and determinants of obesity in China. Lancet Diabetes Endocrinol. (2021) 9:373–92. 10.1016/S2213-8587(21)00045-0 34022156

[5] Pomi AL Pepe G Aversa T Corica D Valenzise M Messina MF Early adiposity rebound: predictors and outcomes. Ital J Pediatr. (2024) 50:98. 10.1186/s13052-024-01671-4 38750561 PMC11094876

[6] Ling J Robbins LB Wen F. Interventions to prevent and manage overweight or obesity in preschool children: a systematic review. Int J Nurs Stud. (2016) 53:270–89. 10.1016/j.ijnurstu.2015.10.017 26582470

[7] Skouteris H Hartley-Clark L Mccabe M Milgrom J Kent B Herring SJ Preventing excessive gestational weight gain: a systematic review of interventions. Obes Rev. (2010) 11:757–68. 10.1111/j.1467-789X.2010.00806.x 20880128

[8] Bleich SN Vercammen KA Zatz LY Frelier JM Ebbeling CB Peeters A. Interventions to prevent global childhood overweight and obesity: a systematic review. Lancet Diabetes Endocrinol. (2018) 6:332–46. 10.1016/S2213-8587(17)30358-3 29066096

[9] Wang Y Zhao L Gao L Pan A Xue H. Health policy and public health implications of obesity in China. Lancet Diabetes Endocrinol. (2021) 9:446–61. 10.1016/S2213-8587(21)00118-2 34097869

[10] Liu N Li H Guo Z Chen X Cheng P Wang B Prevalence and factors associated with overweight or obesity among 2- to 6-year-old children in Hunan, China: a cross-sectional study. Public Health Nutr. (2022) 25:3487–3498. 10.1017/S136898002200012X PMC9991611 35034674

[11] Heymsfield SB Peterson CM Thomas DM Heo M Schuna JJ. Why are there race/ethnic differences in adult body mass index-adiposity relationships? A quantitative critical review. Obes Rev. (2016) 17:262–75. 10.1111/obr.12358 26663309 PMC4968570

[12] Javed A Jumean M Murad MH Okorodudu D Kumar S Somers VK Diagnostic performance of body mass index to identify obesity as defined by body adiposity in children and adolescents: a systematic review and meta-analysis. Pediatr Obes. (2015) 10:234–44. 10.1111/ijpo.242 24961794

[13] Liu WX Chen XY Huang HX Yang GJ Huang XL. Analysis of the current Status and influencing factors of overweight and obesity in preschool children aged 3-6 in Haikou city. Jiangsu Health Manag. (2023) 34:998–1001. [in Chinese]

[14] World Health Organization. WHO child growth standards: length/height-for-age, weight-for-age, weight-for-length, weight-for-height and body mass index-for-age: methods and development. (2006). Available online at: https://www.who.int/publications/i/item/924154693X (Accessed July 07, 2024).

[15] Body mass index-for-age (bmi-for-age). Available online at: https://www.who.int/toolkits/child-growth-standards/standards/body-mass-index-for-age-bmi-for-age (accessed 2024/7/7).

[16] Zong XN Li H Zhang YQ Wu HH. Updated growth standards for Chinese children under 7 years of age. Zhonghua Er Ke Za Zhi. (2023) 61:1103–8. 10.3760/cma.j.cn112140-20230925-00219 37989521

[17] Cole TJ Bellizzi MC Flegal KM Dietz WH. Establishing a standard definition for child overweight and obesity worldwide: international survey. Br Med J. (2000) 320:1240–3. 10.1136/bmj.320.7244.1240 10797032 PMC27365

[18] Yang R Yu J Luo C Qi W Yang D Xue H Correlations and consistency of body composition measurement indicators and BMI: a systematic review. Int J Obes (Lond). (2024). 10.1038/s41366-024-01638-9 39313560

[19] Liu A Byrne NM Kagawa M Ma G Poh BK Ismail MN Ethnic differences in the relationship between body mass index and percentage body fat among Asian children from different backgrounds. Br J Nutr. (2011) 106:1390–7. 10.1017/S0007114511001681 21736824

[20] Liu S Zhang J Ma J Shang Y Ma Y Zhang X Synergistic interaction between bedtime and eating speed in predicting overweight and obesity in Chinese preschool-aged children. Aging (Albany NY). (2019) 11:2127–37. 10.18632/aging.101906 30978174 PMC6503874

[21] Who child growth standards based on length/height, weight and age. Acta Paediatr Suppl. (2006) 95:76–85. 10.1111/j.1651-2227.2006.tb02378.x 16817681

[22] de Onis M Onyango AW Borghi E Siyam A Nishida C Siekmann J. Development of a who growth reference for school-aged children and adolescents. Bull World Health Organ. (2007) 85:660–7. 10.2471/BLT.07.043497 18026621 PMC2636412

[23] Dou QR Zhan HY Cao X Huang CM Huang CC Luo Q Growth curves and reference values for children under 7 years old in hainan province based on the LMS method. Chin J Woman Child Health Res. (2023) 34:17–27. [in Chinese]

[24] Hassapidou M Daskalou E Tsofliou F Tziomalos K Paschaleri A Pagkalos I Prevalence of overweight and obesity in preschool children in Thessaloniki, Greece. Hormones (Athens). (2015) 14:615–22. 10.14310/horm.2002.1601 26188232

[25] Lemelin L Haggerty J Gallagher F. Comparison of three weight classification systems for preschool children in a region of Quebec. Sante Publique. (2013) 25:571–8. 10.3917/spub.135.0571 24418419

[26] Khadilkar VV Khadilkar AV Cole TJ Chiplonkar SA Pandit D. Overweight and obesity prevalence and body mass index trends in Indian children. Int J Pediatr Obes. (2011) 6:e216–24. 10.3109/17477166.2010.541463 21158695

[27] Do LM Tran TK Eriksson B Petzold M Ascher H. Prevalence and incidence of overweight and obesity among Vietnamese preschool children: a longitudinal cohort study. BMC Pediatr. (2017) 17:150. 10.1186/s12887-017-0904-y 28629345 PMC5477312

[28] Ma YN Chen T Wang D Liu MM He QC Dong GH. Prevalence of overweight and obesity among preschool children from six cities of northeast China. Arch Med Res. (2011) 42:633–40. 10.1016/j.arcmed.2011.10.011 22079860

[29] Ji M Tang A Zhang Y Zou J Zhou G Deng J The relationship between obesity, sleep and physical activity in Chinese preschool children. Int J Environ Res Public Health. (2018) 15. 10.3390/ijerph15030527 PMC5877072 29543749

[30] Zhou M Feng X Yong J Li Y Zhang M Page A Lifting the lid on geographic complexity in the relationship between body mass index and education in China. Health Place. (2017) 46:1–5. 10.1016/j.healthplace.2017.02.012 28432911

[31] Dong Y Ma Y Dong B Zou Z Hu P Wang Z Geographical variation and urban-rural disparity of overweight and obesity in Chinese school-aged children between 2010 and 2014: two successive national cross-sectional surveys. BMJ Open. (2019) 9:e025559. 10.1136/bmjopen-2018-025559 30948583 PMC6500219

[32] Jia P Ma S Qi X Wang Y. Spatial and temporal changes in prevalence of obesity among Chinese children and adolescents, 1985–2005. Prev Chronic Dis. (2019) 16:E160. 10.5888/pcd16.190290 31831107 PMC6936667

[33] Zhang X Zhang M Zhao Z Huang Z Deng Q Li Y Geographic variation in prevalence of adult obesity in China: results from the 2013–2014 national chronic disease and risk factor surveillance. Ann Intern Med. (2020) 172:291–3. 10.7326/M19-0477 31658469

[34] Popkin BM. Nutrition, agriculture and the global food system in low and middle income countries. Food Policy. (2014) 47:91–6. 10.1016/j.foodpol.2014.05.001 24932059 PMC4053196

[35] Wang Y Wang L Xue H Qu W. A review of the growth of the fast food industry in China and its potential impact on obesity. Int J Environ Res Public Health. (2016) 13. 10.3390/ijerph13111112 PMC5129322 27834887

[36] Chen JL Esquivel JH Guo J Chesla CA Tang S. Risk factors for obesity in preschool-aged children in China. Int Nurs Rev. (2018) 65:217–24. 10.1111/inr.12371 28326536

[37] Piernas C Wang D Du S Zhang B Wang Z Su C The double burden of under- and overnutrition and nutrient adequacy among Chinese preschool and school-aged children in 2009–2011. Eur J Clin Nutr. (2015) 69:1323–9. 10.1038/ejcn.2015.106 26130296 PMC4668216

[38] Xiao Y Qiao Y Pan L Liu J Zhang T Li N Trends in the prevalence of overweight and obesity among Chinese preschool children from 2006 to 2014. PLoS One. (2015) 10:e0134466. 10.1371/journal.pone.0134466 26267264 PMC4534378

[39] Garrido-Miguel M Oliveira A Cavero-Redondo I Alvarez-Bueno C Pozuelo-Carrascosa DP Soriano-Cano A Prevalence of overweight and obesity among European preschool children: a systematic review and meta-regression by food group consumption. Nutrients. (2019) 11. 10.3390/nu11071698 PMC6682909 31340602

[40] Ricciardelli LA Mccabe MP. Children’s body image concerns and eating disturbance: a review of the literature. Clin Psychol Rev. (2001) 21:325–44. 10.1016/S0272-7358(99)00051-3 11288604

[41] Song Y Wang HJ Ma J Wang Z. Secular trends of obesity prevalence in urban Chinese children from 1985 to 2010: gender disparity. PLoS One. (2013) 8:e53069. 10.1371/journal.pone.0053069 23308137 PMC3540080

[42] Lombardo M Feraco A Armani A Camajani E Gorini S Strollo R Gender differences in body composition, dietary patterns, and physical activity: insights from a cross-sectional study. Front Nutr. (2024) 11:1414217. 10.3389/fnut.2024.1414217 39055386 PMC11271261

[43] Li J Lei J Wen S Zhou L. Sex disparity and perception of obesity/overweight by parents and grandparents. Paediatr Child Health. (2014) 19:e113–6. 10.1093/pch/19.7.e113 25332680 PMC4173911

[44] Sijtsma A Sauer PJ Corpeleijn E. Parental correlations of physical activity and body mass index in young children–he gecko drenthe cohort. Int J Behav Nutr Phys Act. (2015) 12:132. 10.1186/s12966-015-0295-0 26453436 PMC4599029

[45] Mei H Guo S Lu H Pan Y Mei W Zhang B Impact of parental weight status on children’s body mass index in early life: evidence from a Chinese cohort. BMJ Open. (2018) 8:e18755. 10.1136/bmjopen-2017-018755 PMC6020987 29921677

[46] Xu RY Zhou YQ Zhang XM Wan YP Gao X. A two-year study of parental obesity status and childhood obesity in China. Nutr Metab Cardiovasc Dis. (2019) 29:260–7. 10.1016/j.numecd.2018.11.004 30642789

[47] Engeli S Lehmann AC Kaminski J Haas V Janke J Zoerner AA Influence of dietary fat intake on the endocannabinoid system in lean and obese subjects. Obesity (Silver Spring). (2014) 22:E70–6. 10.1002/oby.20728 24616451

[48] Gardner KR Sapienza C Fisher JO. Genetic and epigenetic associations to obesity-related appetite phenotypes among African-American children. Pediatr Obes. (2015) 10:476–82. 10.1111/ijpo.12010 25779370

[49] Woo BJ Locks LM Cheng ER Blake-Lamb TL Perkins ME Taveras EM. Risk factors for childhood obesity in the first 1,000 days: a systematic review. Am J Prev Med. (2016) 50:761–79. 10.1016/j.amepre.2015.11.012 26916261

[50] Zhang X Liu E Tian Z Wang W Ye T Liu G High birth weight and overweight or obesity among Chinese children 3-6 years old. Prev Med. (2009) 49:172–8. 10.1016/j.ypmed.2009.07.013 19632265

[51] Liao P Wang WJ Yu HT Zang JJ Qian NS He X Differences and correlation analysis of birth weight and overweight/obesity in Shanghai twin cohort. Twin Res Hum Genet. (2021) 24:29–36. 10.1017/thg.2021.2 33645497

[52] Wang Q Yang M Deng X Wang S Zhou B Li X Explorations on risk profiles for overweight and obesity in 9501 preschool-aged children. Obes Res Clin Pract. (2022) 16:106–14. 10.1016/j.orcp.2022.02.007 35277363

[53] Dietz WH. Critical periods in childhood for the development of obesity. Am J Clin Nutr. (1994) 59:955–9. 10.1093/ajcn/59.5.955 8172099

[54] Chatterjee S Ouidir M Tekola-Ayele F. Pleiotropic genetic influence on birth weight and childhood obesity. Sci Rep. (2021) 11(48). 10.1038/s41598-020-80084-9 PMC7794220 33420178

[55] Zong XN Li H Zhang YQ. Family-related risk factors of obesity among preschool children: results from a series of national epidemiological surveys in China. BMC Public Health. (2015) 15:927. 10.1186/s12889-015-2265-5 26386823 PMC4575782

